# Sex-dependent effects of chronic jet lag on circadian rhythm and metabolism in mice

**DOI:** 10.1186/s13293-024-00679-z

**Published:** 2024-12-05

**Authors:** Tiantian Ma, Ryohei Matsuo, Kaito Kurogi, Shunsuke Miyamoto, Tatsumi Morita, Marina Shinozuka, Fuka Taniguchi, Keisuke Ikegami, Shinobu Yasuo

**Affiliations:** Laboratory of Regulation in Metabolism and Behavior, Faculty of Agriculture, Kyushu University, 744 Motooka, Nishi-ku, Fukuoka, 819-0395 Japan

**Keywords:** Sex difference, Circadian rhythm, Metabolism, Glucose intolerance, Testosterone

## Abstract

**Background:**

The circadian clock integrates external environmental changes into the internal physiology of organisms. Perturbed circadian clocks due to misaligned light cycles increase the risk of diseases, including metabolic disorders. However, the effects of sex differences in this context remain unclear.

**Methods:**

Circadian misalignment was induced by a chronic jet lag (CJL) shift schedule (light-on time advanced by 6 h every 2 days) in C57BL/6N male and female mice. Core body temperature and activity rhythms were recorded using a nano tag, and the gene expression rhythms of clock and clock-controlled genes in the liver and adrenal glands were analyzed using qPCR. Glucose metabolism and insulin response were evaluated using glucose tolerance, insulin sensitivity, and glucose response assays. Castration and testosterone replacement were performed to assess the fundamental role of testosterone in male phenotypes under CJL.

**Results:**

Under CJL treatment, male mice exhibited increased weight gain, whereas females exhibited decreased weight gain compared to that of the respective controls. CJL treatment induced a lower robustness of circadian rhythms in core body temperature and a weaker rhythm of clock gene expression in the liver and adrenal glands in females, but not in males. Only male mice exhibited glucose intolerance under CJL conditions, without the development of insulin resistance. Castrated mice without testosterone exhibited decreased weight gain and reduced robustness of body temperature rhythm, as observed in intact females. Testosterone replacement in castrated mice recovered the CJL-induced weight gain, robustness of temperature rhythm, and glucose intolerance observed in intact males.

**Conclusions:**

Significant sex-based differences were observed in circadian clock organization and metabolism under CJL. Testosterone plays a crucial role in maintaining the circadian clock and regulating CJL metabolism in males.

**Supplementary Information:**

The online version contains supplementary material available at 10.1186/s13293-024-00679-z.

## Abstract (significance)

This study investigated the effects of chronic jet lag (CJL) on male and female mice, revealing significant differences in their responses to frequent light-dark shifts that induce circadian disruptions. Male mice exhibited increased body weight gain under CJL conditions, while in female mice, it was decreased. CJL treatment weakened the circadian rhythms of body temperature and clock gene expression in peripheral organs in females, but not in males. Only male mice developed glucose intolerance without developing insulin resistance. When male mice were castrated, they experienced reduced weight gain and changes in their circadian rhythms similar to that of females, but replacing testosterone restored their weight gain and glucose intolerance. Overall, this research highlights significant differences between the sexes in regard to how CJL impacts the circadian clock and metabolism, emphasizing the important role of testosterone in regulasting these processes in males.

**Supplementary Information:**

The online version contains supplementary material available at 10.1186/s13293-024-00679-z.

## Highlights

Chronic jet lag increased weight gain in male mice and decreased it in female mice.

Female mice exhibited vulnerable circadian rhythms following chronic jet lag.

Male mice exhibited glucose intolerance under chronic jet lag.

Testosterone is crucial for maintaining circadian rhythm and metabolism in males.

**Supplementary Information:**

The online version contains supplementary material available at 10.1186/s13293-024-00679-z.

## Background

The circadian clock plays an essential role in orchestrating the circadian rhythms of behavioral and physiological outputs within approximately 24 h. Circadian rhythm is regulated by the central pacemaker in the suprachiasmatic nucleus (SCN), along with peripheral circadian oscillators in various tissues and organs of the body, composing a hierarchical system [1]. The molecular clock involves transcription-translation feedback loops that consist of clock genes [2]. The core clock proteins BMAL1 and CLOCK form a heterodimer that initiates the transcription of the *Pers* and *Crys* genes by binding to E-box sequences. As PERs and CRYs proteins accumulate during the day, they are translocated into the nucleus and interact with the BMAL1-CLOCK complex to repress their transcription. After rapid degradation, negative-feedback repression was relieved following a new 24-hour cycle transcribed by the BMAL1-CLOCK complex the next morning. In addition to *Pers* and *Crys*, the BAML1-CLOCK complex also activates the nuclear receptors REV-ERBα and REV-ERBβ that bind to ROR response elements in the promoters of *Bmal1*. Additionally, there is a regulatory loop driven by CLOCK-BMAL1 that involves the PAR-bZip transcription factor D-box binding protein (DBP). CLOCK-BMAL1 binds to E-box elements in the promoter region of *Dbp* and activates its transcription in a circadian manner. In turn, DBP binds to the D-box regulatory elements in the promoters of clock-controlled genes and coordinates circadian expression.

The molecular clock also governs the expression of numerous clock-controlled genes that are involved in a wide range of physiological processes. The circadian clock is tightly coupled with metabolic pathways at the molecular level, particularly in the liver. For example, REV-ERBα regulates the activity rhythms of SREBP1, a master transcription factor of lipid metabolism in the liver. The rhythmic nuclear accumulation of SREBP1 is phase-shifted, leading to misregulation of *Srebp1* target genes involved in lipid metabolism in the liver of *Rev-erbα* knockout mice [3]. PER2 interacts with the nuclear receptor PPARα, which is a key regulator of fatty acid oxidation, serves as a coregulator of nuclear receptor-mediated transcription, and influences PPARα-regulated metabolic pathways [4]. CLOCK binds to E-box elements within the first intron of *Gys2* and rhythmically activates its transcription in the liver. Mutation of *Clock* dampens circadian oscillations of *Gys2* mRNA and protein levels, leading to corresponding oscillations in hepatic glycogen levels [5]. Accumulating evidence has demonstrated that the circadian clock regulates daily rhythms in the expression of numerous genes involved in glycogen synthesis and degradation, gluconeogenesis, lipid metabolism, bile acids, and cholesterol metabolism [6, 7].

Irregular light-dark cycles, such as those caused by shift work, irregular sleep schedules, and frequent travel across time zones, can disrupt the circadian clock system. Irregular cycles are associated with the risk of numerous diseases, including diabetes and obesity [8, 9]. In a human experiment, circadian misalignment protocols based on using two 8-d laboratory schedules in healthy men induced glucose tolerance decreases in the evening, along with decreased insulin sensitivity and pancreatic β-cell function [10]. The mechanisms underlying this association have been studied using animal models with aberrant lighting conditions, such as constant light, dim light at night [11], shortened periods of light-dark cycles (e.g., 10 h light – 10 h dark) [8], or chronic jet lag (CJL) [12]. The CJL protocol involves frequent shifts of advancing light/dark cycles by 6–8 h every 2–7 days and induces dysregulation of circadian rhythms, metabolism, and behaviors in rodents [12–15]. CJL serves as a powerful method to induce physiological and behavioral consequences of chronic circadian misalignment. For example, CJL with 6 h advances of light-dark cycles every two days for 8 weeks increased anxiety and depression-like behaviors and decreased motivation for food reward in mice [13]. The light regimen for 30 days also increases body weight gain, adipose tissue mass, and circulating triglycerides [15] and induces desynchronized activity-rest rhythms [14] in mice. Most studies utilizing these protocols have focused on male mice that consistently exhibit signs of increased weight and glucose intolerance without changes in total daily food intake under aberrant lighting conditions [8, 11, 12]. Therefore, these findings highlight the need for further research investigating the sex-specific effects of CJL on metabolic health.

Circadian rhythms in mammals are highly sex-dependent. The SCN and other brain regions contain androgen and estrogen receptors that regulate activity rhythms in a sex-dependent manner. In males, castration leads to altered circadian rhythms, including longer circadian periods under dim red light and imprecise activity onset timing, which are restored by testosterone replacement [16, 17]. Ovariectomized female mice exhibit decreased total activity levels and impaired precision of the daily onset of activity rhythms without changing the circadian period, and estradiol treatment restores these reduced activity rhythms [18, 19]. Light resetting of wheel-running activity rhythms also leads to sexual dimorphism. Male mice display greater precision in activity onset, whereas females undergo sexual cycle-dependent changes in activity duration, called scalloping [20]. Additionally, the PER2::LUC bioluminescence rhythm is phase-delayed in the female pituitary gland and liver compared to that in males [20]. Consequently, the effects of disturbances in regular light-dark cycles differ between males and females. Recent laboratory studies [21] investigating circadian misalignment in humans have demonstrated sex differences in metabolic conditions. Following a 12-hour phase shift that caused circadian misalignment, leptin levels in men increased, whereas women experienced a decrease in leptin coinciding with an increase in ghrelin. This results in changes in the types of food cravings between the sexes, such as elevated cravings for energy-dense and savory foods in men [21]. However, observational studies have presented conflicting results, indicating that shift work exhibits either a stronger [22, 23] or weaker [24, 25] correlation with obesity and/or metabolic syndrome in women than in men. The discrepancies between these studies could be attributed to systematic variations, such as differences in shift schedules, food intake patterns, and food preferences under stress. To determine the biological impact of circadian misalignment, animal studies based on standardized lighting schedules and food conditions are necessary.

Therefore, the objective of this study was to elucidate specific alterations in the circadian clock and metabolic processes in both females and males under conditions of circadian misalignment and to discern inherent differences between the sexes. A recent study using male and female C57BL/6J mice fed a high-fat diet (HFD) reported that female mice were more resilient than male mice in terms of behavioral and transcriptional rhythmicity [26]. C57BL/6 substrains, C57BL/6J from Jackson Laboratories, and C57BL/6N from the National Institutes of Health possess several different genetic variants. With a functional deletion of exons 7–11 in the gene encoding nicotinamide nucleotide transhydrogenase [27], the C57BL/6J strain is more vulnerable to HDF-induced obesity [28, 29] and more glucose intolerant than C57BL/6N mice [30]. Thus, we performed circadian misalignment using a CJL shift schedule in C57BL/6N male and female mice, followed by comparison with C57BL/6J mice under standard diet conditions. As C57BL/6N mice displayed higher sexual differences in body weight under CJL, we used this substrain to analyze the circadian clock system and metabolism, including metabolic gene expression in the liver as a major metabolic organ that is sensitive to CJL, glucose intolerance, and insulin response to glucose. We then performed castration and testosterone replacement to elucidate the role of testosterone in sex differences. Overall, the results indicate that female clocks are more vulnerable to CJL than males under a standard diet, whereas glucose tolerance worsens only in males. Testosterone plays an essential role in the male phenotype under CJL.

## Methods

### Animals

Six-week-old male and female C57BL/6N and C57BL/6J mice were purchased from Japan SLC (Shizuoka, Japan). Upon arrival, the mice were housed in standard cages (175 × 245 × 125 mm) in groups of four. They were fed a commercial diet (MF; Oriental Yeast, Tokyo, Japan) comprising crude protein (23.2%), crude fat (4.9%), crude ash (5.9%), crude fiber (3.3%), and moisture (8.1%), providing 355.7 kcal, and drinking water *ad libitum*. The cages were placed in a box in a room at 24 ± 1 °C. They were maintained under 12 h of light and 12 h of darkness (12 L:12D) for at least one week before the start of the experiments. The light intensity on the heads of the animals was approximately 70 lx. All animal experiments were conducted in accordance with the Guidelines for Animal Experiments of the Faculty of Agriculture at Kyushu University and the Law (No. 105) and Notification (No. 6) of the Japanese Government. All experiments were approved by the Animal Care and Use Committee of Kyushu University (approval numbers: A21-398 and A23-217).

### CJL treatment

**Fig. 1:**
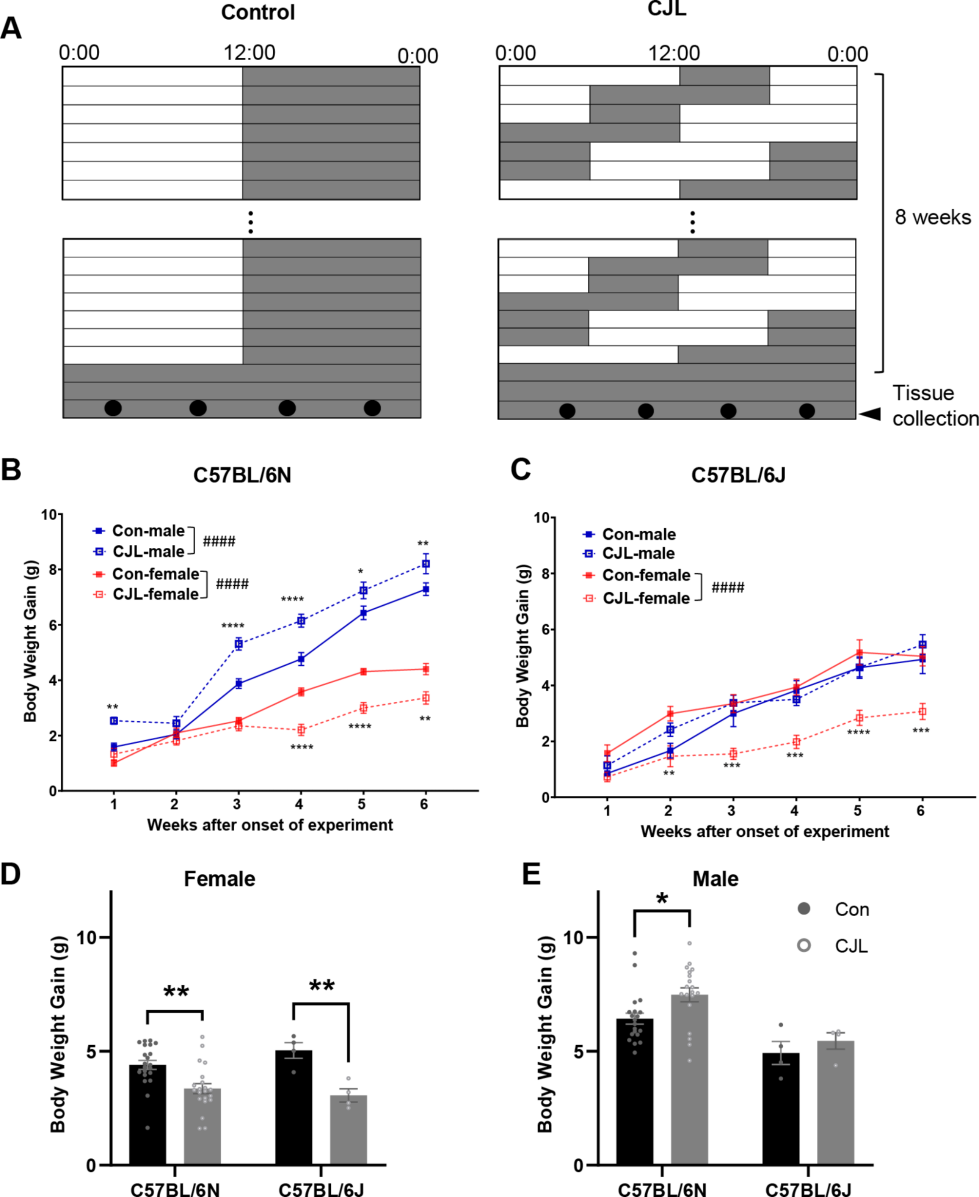
Sex-dependent changes in body weight gain under CJL in C57BL/6N and C57BL/6J mice. (**A**) Schematic illustration of light schedules in control and chronic jet lag (CJL) groups. White and gray colors represent the light and dark phases, respectively. (**B**) Body weight gain of C57BL/6N mice under CJL. Data are presented as means ± standard errors of the means (S.E.M) (*n* = 21 per group in each sex, combined data of mice subjected to four-time points sampling and nano tag recording). (**C**) Body weight gain of C57BL/6J mice under 6 weeks CJL treatment (*n* = 4 per group in each sex). (**D, E**) Comparison of body weight gain of C57BL/6N and C57BL/6J female (**D**) and male (**E**) mice. The gain on week 6 of CJL treatment is shown. Two-way ANOVA followed by Šidák’s multiple comparison test, *#### p* < 0.0001, * p < 0.05, ** p < 0.01, *** p < 0.001, **** p < 0.0001

C57BL/6N mice (male: *n* = 32, female: *n* = 32) were housed in groups of four per cage. Half of the mice were designated as control mice and housed under 12 L:12D. The other half of the mice were designated as the CJL group, in which lights on and off times were advanced by 6 h every 2 days for 8 weeks, according to a published method [13]. The CJL schedule is shown in Fig. 1A. Body weight was measured weekly during treatment. Food intake was measured in the 5th, 6th, and 7th weeks. Computed tomography (CT) scan was performed at the beginning of CJL and at week 6 using Cosmo Scan FX (Rigaku, Tokyo, Japan) with the following parameters: tube voltage, 90 kV; tube current, 88 µA; exposure time, 2 min; resolution, 10 μm isotropic. Micro-CT images were evaluated in three dimensions. During scanning, the mice were anesthetized with isoflurane. The open field test (OFT) was performed in the 6th week to evaluate anxiety-like behavior. Briefly, each mouse was placed at the center of an open field box (40 × 40 × 40 cm, 40 lx, central area: 10 × 10 cm), and the behavior of each mouse was analyzed for 5 min using a video tracking system (ANY-maze; Stoelting, Illinois, United States). C57BL/6J mice (male: *n* = 8, female: *n* = 8) were separated into control and CJL groups in a similar manner as C57BL/6N mice, and their body weights were recorded every week.

Eight weeks after CJL, all C57BL/6N mice were transferred to constant darkness (DD) for 2 days and then euthanized using isoflurane at 2:00, 8:00, 14:00, and 20:00 based on the lighting conditions prior to the transfer to DD (0:00 was defined as the light onset on the prior day, four animals in each group per time point). The timing was determined, as modest individual variations of circadian phases are expected within 3 days of DD following CJL [12]. Brains were frozen on dry ice and stored at − 80°C. Blood was centrifuged at 3,000 × *g* for 10 min at 4℃, and plasma was stored at − 80°C. Liver fragments and adrenal glands were collected using RNAlater reagent (Ambion, TX, USA). In female mice, vaginal cytology was performed after euthanasia to confirm the estrous phase, and no significant bias between the groups was confirmed.

### Nano tag recording

Another cohort of C57BL/6N mice (male: *n* = 10, female: *n* = 10) was used to measure core body temperature and locomotor activity rhythms using a nano tag (Kissei Comtec Co., Matsumoto, Japan). The mice were maintained under control and CJL conditions (five animals in each group and sex) for eight weeks, followed by DD conditions. One day before week 8 of CJL treatment, a nano tag was implanted into the abdominal cavity under anesthesia using an isoflurane inhalation anesthesia device (Natsume Seisakusho Co., Ltd., Tokyo, Japan). After surgery, cephalexin (15 mg/kg) dissolved in saline was injected subcutaneously. Core body temperature and activity levels were recorded every 5 min for one week under CJL (week 8) and the following week under DD. The data for the LD and DD conditions were analyzed separately by chi-square periodogram analysis using ClockLab software (Actimetrics, Wilmette, IL, USA, version 6.1.15). The circadian period and rhythm robustness which is power of the chi-square periodogram (Qp value) were used to evaluate the effect of CJL on circadian rhythmicity.

### qPCR

Total RNA was extracted from the tissues using ISOGEN II (Nippon Gene, Tokyo, Japan) according to the manufacturer’s protocol. cDNA was synthesized using 1 µg of total RNA and a Primer Script & RT reagent kit with gDNA Eraser (Takara, Kusatsu, Japan) according to the manufacturer’s protocol. qPCR was performed using Stratagene Mx3000P (Agilent Technologies, Santa Clara, USA) with a denaturation step at 95°C for 30 s, 40 cycles of amplification at 95°C for 5 s, and a primer-specific annealing temperature for 30 s. Primer sequences and annealing temperatures are listed in Supplementary Table 1. Each mRNA level was calculated using threshold cycles for the amplification of unknown samples and was compared with those of the four concentrations of standard cDNAs. The calculated levels were normalized to the expression levels of *36b4*. Melting curve analysis was performed for each gene to validate the specificity of the PCR conditions.

### Measurements of insulin and glucose in plasma, glycogen, and fat in liver

Plasma glucose and insulin concentrations were measured using a Glucose CII Test Wako Kit (Fujifilm Wako Pure Chemical Co., Osaka, Japan) and an Ultra-Sensitive Mouse Insulin ELISA Kit (Morinaga Institute of Biological Science, Yokohama, Japan), respectively, according to the manufacturer’s protocol.

Liver glycogen was stained using the periodic acid-Schiff staining (PAS) method using a commercially available kit (Muto Pure Chemicals, Tokyo, Japan). Briefly, liver tissues were embedded in Tissue-Tek O.C.T. compound (Sakura Tissue Tek, Tokyo, Japan) and frozen in liquid nitrogen. The sections were fixed in 4% paraformaldehyde and soaked sequentially in 1% periodic acid solution for 5 min, Schiff reagent for 30 min at 37°C, sulfurous acid solutions for 5 min, and Carrazi hematoxylin for 15 min. Liver fat accumulation was assessed using Oil Red O (Sigma-Aldrich, St. Louis, USA) staining of frozen liver sections that was performed according to a previously described protocol [31]. Briefly, the sections were fixed in 4% paraformaldehyde and soaked in 60% isopropanol for 15 s and then Oil Red O working solution for 30 min, rinsed in 60% isopropanol for 15 s, washed with distilled water, and mounted on slides. Microscope images (689.55 μm x 517.16 μm for PAS staining, 355.56 μm x 266.67 μm for Oil Red O staining) were acquired from sections. Optical densities for PAS staining and stained areas for Oil Red O staining were analyzed using ImageJ (National Institutes of Health). The data are expressed as the percentage of the maximum value of all animals.

### Glucose and insulin tolerance tests

A cohort of C57BL/6N mice (male: *n* = 8, female: *n* = 8) was used to evaluate glucose and insulin tolerance under CJL, followed by confirmation of insulin tolerance using two other cohorts (male: *n* = 8, female: *n* = 8 for each cohort) with different doses of insulin. The fourth cohort (males, *n* = 6; females, *n* = 6) was used to evaluate insulin response to glucose. For all cohorts, male and female mice were separated into control and CJL groups and subjected to the respective light-dark cycles, as described above.

For the first cohort on the first day of week 7 under CJL, the mice in the control and CJL groups were fasted overnight one day before testing. On the day of the experiment, tail vein blood glucose levels were measured after starvation and recorded using a glucometer (Sanwa Kagaku Kenkyusho, Nagoya, Japan). Next, 2 g/kg glucose was intraperitoneally injected at 2:00, and tail vein blood glucose levels were measured and recorded sequentially for 90 min (15, 30, 60, and 90 min) using a glucometer. Using the temporal changes in glucose levels, the incremental area under the curve (iAUC) was calculated for each animal as a glucose tolerance parameter. For the insulin resistance test on the last day of week 7 under CJL, the mice were starved for 2 h, and the primary glucose level was measured at 6:00. Insulin (0.75 units/kg body weight) was injected intraperitoneally. Blood glucose levels in the tail vein were measured 20, 40, 60, and 120 min after insulin injection. For the second and third cohorts, insulin tolerance tests using different doses of insulin (0.5 or 1 unit/kg) were performed simultaneously as in the first cohort. Using temporal changes in glucose levels, the *k*ITT was calculated from the linear slope of the plasma glucose concentration curve between 0 and 20 min as a parameter of insulin sensitivity. To evaluate the insulin response to glucose on the 8th week of CJL treatment, mice in the fourth cohort were fasted overnight one day before testing. Plasma was collected before and 20 min after glucose intraperitoneal injection (2 g/kg) and stored at − 80°C for insulin measurement.

### Castration and testosterone replacement

Male C57BL/6N mice were acclimated for 7 days. Prior to CJL treatment, the animals were bilaterally castrated. Surgeries were performed under isoflurane anesthesia. The testes were externalized via laparotomy and removed after clamping the testicular artery. Silicone tubes (inner diameter = 2.0 mm, outer diameter = 3.0 mm; Kaneka, Osaka, Japan) were filled with 100% testosterone propionate or 100% cholesterol and sealed with silicone adhesive. The tubes were 12 mm long, with an additional 3 mm on each end of the adhesive. In this method, testosterone constantly leaks from the tubes [32]. One day before implantation, the tubes were washed with 70% ethanol, primed in a saline solution at 37 °C overnight, and then implanted subcutaneously under isoflurane anesthesia. Castrated mice implanted with testosterone- or vehicle-containing tubes were subjected to control (12 L:12D) or CJL treatment as described above for six weeks, followed by DD conditions. Body weight was recorded weekly. Nano tags were implanted at week 5, and body temperature and activity rhythms during the CJL and DD conditions were recorded. CJL light was restarted the following week, and a glucose tolerance test was performed two weeks later. One week after the glucose tolerance test, all mice were euthanized using isoflurane at 16:00. Trunk blood was collected in heparinized tubes and centrifuged at 3,000 × *g* for 10 min at 4 °C to obtain plasma samples for testosterone measurement.

### Testosterone measurement

The plasma sample was transferred to a glass tube and spiked with an isotope-labelled internal standard solution containing testosterone-^13^C_3_. Testosterone was extracted with 4 mL of methyl tert-butyl ether. Following evaporation, the extract was dissolved in methanol (0.5 mL) and diluted with distilled water (1 mL). The sample was applied to an OASIS MAX cartridge that had been previously conditioned with methanol (3 mL) and distilled water (3 mL). The cartridge was then washed with 1 mL of distilled water, 1 mL of methanol/distilled water/acetic acid (45:55:1, v/v/v), and 1 mL of 1% pyridine solution. Finally, the steroids were eluted with 1 mL of methanol/ pyridine (100:1, v/v).

After evaporation, the residue was reacted with 50 µL of mixed solution (80 mg of 2-methyl-6-nitrobenzoic anhydride, 20 mg of 4-dimethylaminopyridine, 40 mg of picolinic acid, and 10 µL of triethylamine in 1 mL of acetonitrile) for 30 min. Subsequently, the sample was dissolved in 0.5 mL of ethyl acetate/hexane/acetic acid (15:35:1, v/v/v), and the mixture was applied to an InertSep SI cartridge that had been previously conditioned with 3 mL of acetone and 3 mL of hexane. The cartridge was washed with 1 mL of hexane and 2 mL of ethyl acetate/hexane (3:7, v/v). Testosterone was eluted with 2.5 mL of acetone/hexane (7:3, v/v). After evaporation, the residue was dissolved in 0.1 mL of acetonitrile/distilled water (2:3, v/v), and the solution was subjected to LC-MS/MS. The SRM transitions were *m/z* 394.3/253.2 for T and *m/z* 397.2/256.2 for T-^13^C_3_, respectively. The measurement range was 2.9–14,285 pg/mL.

### Statistical analysis

Weekly changes in body weight were analyzed using a two-way ANOVA, followed by Šidák’s multiple comparison test. The effects of CJL were analyzed in each sex. The results of the behavioral tests were analyzed using one-way ANOVA or unpaired *t*-test. Values were considered significantly different at *p* < 0.05. In the castration and testosterone replacement experiments, body weight changes were analyzed using two-way ANOVA, followed by Šidák’s multiple comparison test.

Rhythmic variations of clock genes and metabolic gene expression in the liver and adrenal gland as well as plasma insulin, glucose, and liver glycogen levels were analyzed by two-way ANOVA based on treatment and sampling time points (2:00, 8:00, 14:00, 20:00). Cosinor analysis was also performed based on linear harmonic regression using CircWave software (Roelof Hut, University of Groningen, version 1.4) with 0.05 for an assumed period of 24 h. This analysis allowed us to estimate the significance of circadian variations, acrophases as centers of gravity, and the amplitude from the peak to the middle of the fitted cosinor wave. Although both control and CJL mice were euthanized for tissue sampling based on the lighting conditions prior to the transfer to DD, the onsets of free-running rhythms of locomotor activity differed between control and CJL. Thus, we determined the onset of locomotor activity using ClockLab software and defined the averaged time in a group as circadian time (CT) 12. In the control male and female groups, 2:00, 8:00, 14:00, and 20:00 correspond to CT 2, CT 8, CT 14, and CT 20, respectively. In the CJL female groups, these time points correspond to CT 7, CT 13, CT 19, and CT 1, respectively, while in the CJL male groups, they correspond to CT 18, CT 24, CT 6, and CT 12. The data are presented with CTs.

## Results

### Sex-dependent changes in body weight gain under CJL

Mice exposed to CJL exhibited sex-dependent effects on body weight gain. Male mice exhibited increased weight gain compared to controls, whereas female mice exhibited decreased weight gain in the C57BL/6N strain (*p* < 0.0001, Fig. 1B). CJL exerted no significant effect on food intake in either sex (Supplementary Fig. 1A). Total fat mass gain was not altered by 6 weeks of CJL treatment in either male or female mice (Supplementary Fig. 1B). To compare the effects of CJL on body weight between C57BL/6N and C57BL/6J mice, C57BL/6J mice were subjected to CJL treatment. These results were similar to the sex-dependent outcomes observed in C57BL/6N mice; however, the observed variance in weight change was only present in C57BL/6J female mice (*p* < 0.0001, Fig. 1C). When the body weights at week 6 were compared between substrains in each sex, females exhibited reduced body weight gain irrespective of substrains (*p* = 0.0016 for C57BL/6N, *p* = 0.0092 for C57BL/6J, Fig. 1D), but males exhibited increased gains only in C57BL/6N mice (*p* = 0.0156, Fig. 1E). Therefore, we used C57BL/6N mice to analyze the sex-dependent effects of CJL on the circadian clock and metabolism in the following experiments.

### Reduced robustness of body temperature rhythms under CJL occurred only in females

**Fig. 2:**
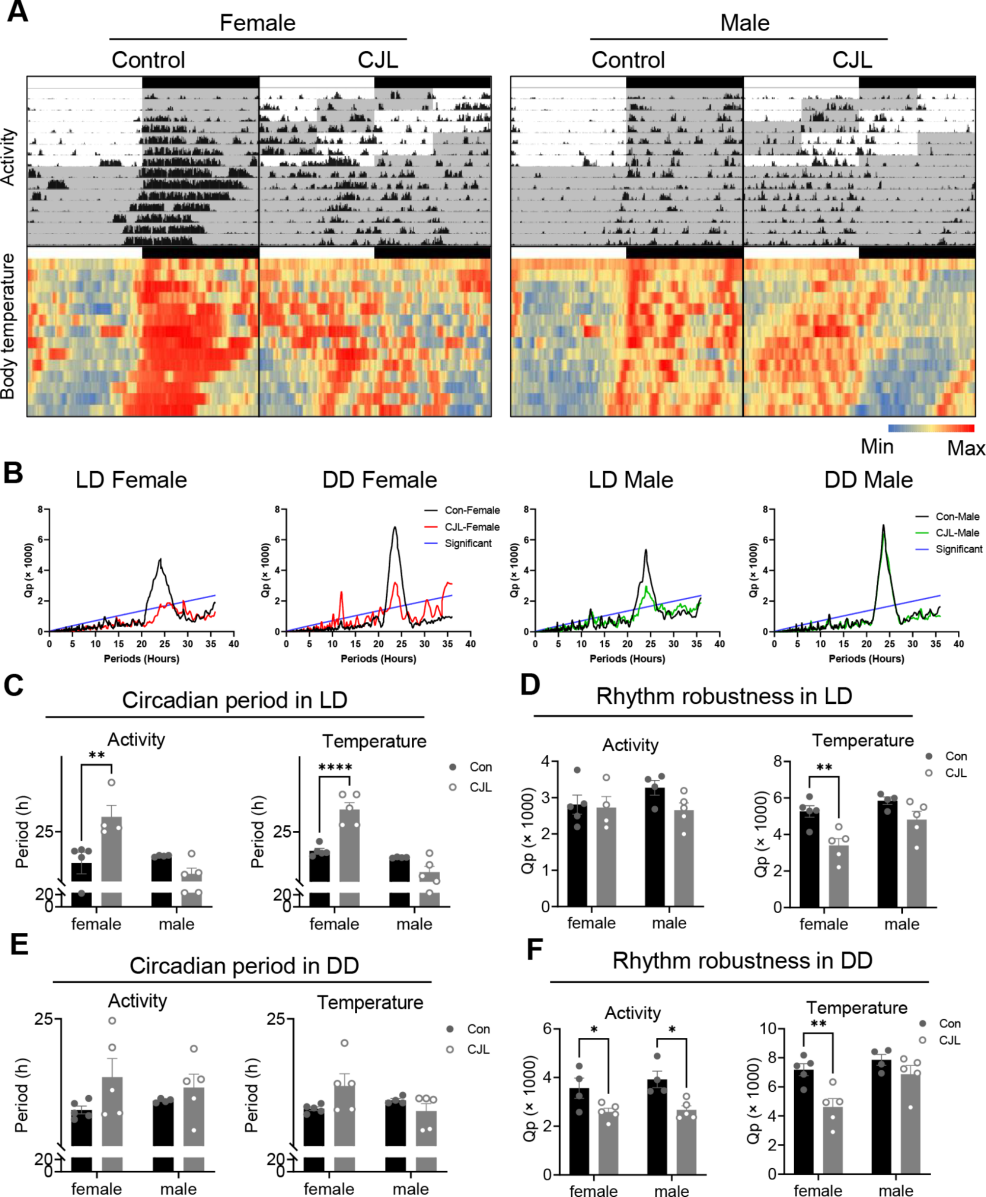
Sex-dependent changes in body temperature and activity rhythms under CJL. (**A**) Actograms of locomotor activity and body temperature rhythms under control or chronic jet lag (CJL) conditions in female and male C57BL/6N mice. (**B**) Chi-square periodogram based on body temperature rhythms under light-dark cycles (LD, control or CJL conditions) or following constant darkness (DD). The blue line is the significance level (*p* = 0.001). (**C–F**) The circadian period (**C, E**) and rhythm robustness (Qp) (**D, F**) of activity and body temperature rhythms of female and male mice under LD (**C, D**) and following DD (**E, F**), based on chi-square periodogram. Data are presented as means ± standard errors of the means (S.E.M). Two-way ANOVA followed by Šidák’s multiple comparison test, * p < 0.05, ** p < 0.01, **** p < 0.0001

During CJL and the subsequent DD conditions, significant circadian activity and body temperature rhythms were sustained in both sexes (Fig. 2A and B). The circadian periods of activity (*p* = 0.0039) and body temperature (*p* < 0.0001) were significantly lengthened by CJL treatment in female mice but not in male mice (Fig. 2C). The rhythm robustness (Qp values) of body temperature was significantly reduced under CJL in females (*p* = 0.0041, Fig. 2D). Under DD conditions after CJL treatment, the circadian periods of activity and body temperature rhythms exhibited no significant differences compared to that of the control in both male and female mice (Fig. 2E). The robustness of activity rhythms decreased in both males (*p* = 0.0100) and females (*p* = 0.0434), and the robustness of body temperature rhythms was significantly reduced under CJL in females (*p* = 0.0048), but not in males (Fig. 2F). We also performed an OFT to analyze behavioral changes in male and female mice treated with CJL. Male mice exhibited no changes compared to control mice, whereas female mice spent less time in the center (*p* = 0.0003), indicating anxiety-like behavior (Supplementary Fig. 2).

### Weakened rhythmicity of clock genes expression in organs under CJL in females

**Fig. 3:**
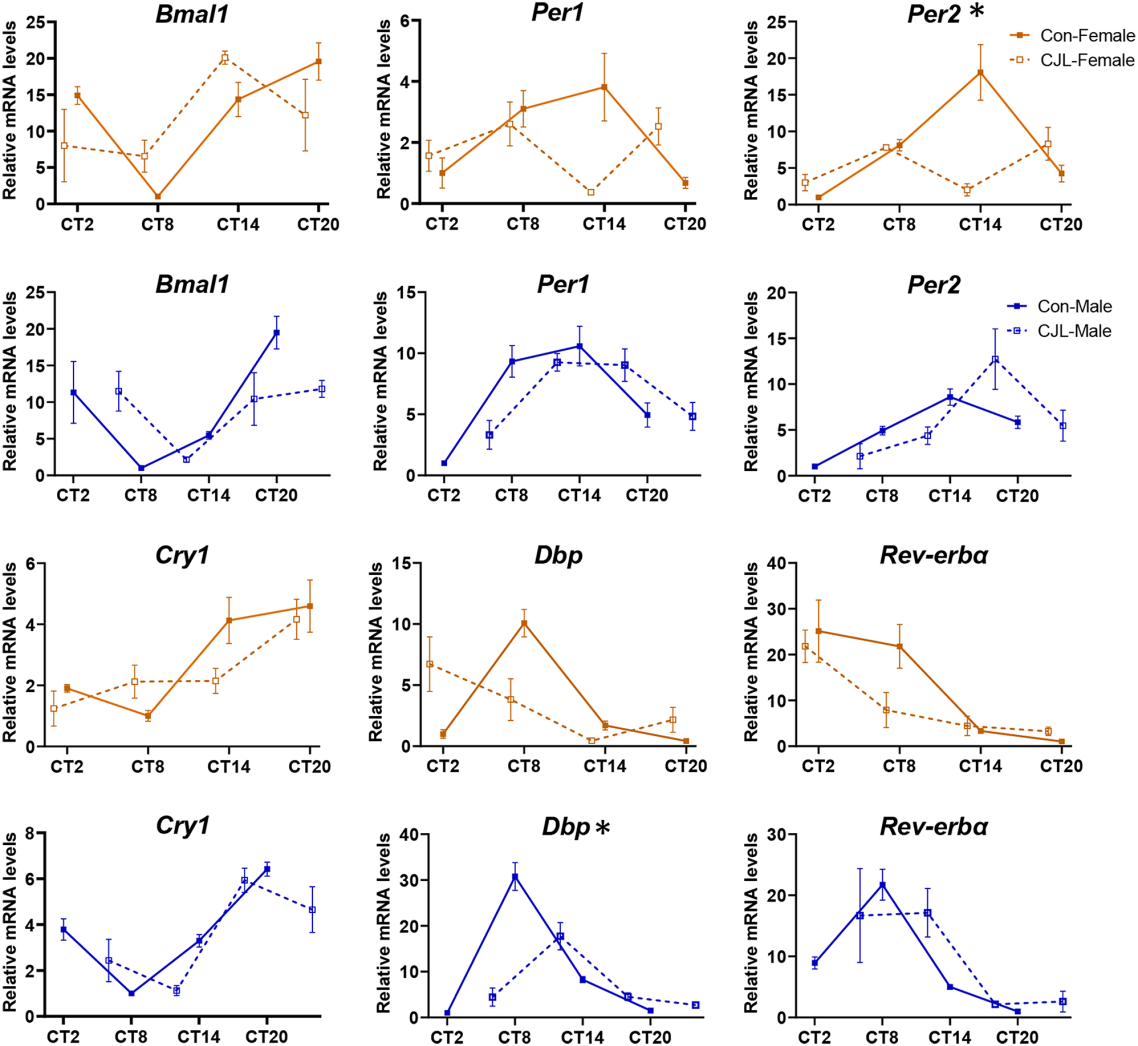
Effect of CJL on rhythmic expression of clock genes expression in the liver. Expression of circadian clock genes *Bmal1*, *Per1*, *Per2*, *Cry1*, *Rev-erbα*, as well as clock-controlled gene (*Dbp*) in the livers of female and male C57BL/6N mice in control or chronic jet lag (CJL) groups. Data are presented as means ± standard errors of the means (S.E.M) (*n* = 4 per time point). Two-way ANOVA, the main effects of CJL treatments, *p < 0.05, based on sampling time points (2:00, 8:00, 14:00, and 20:00). Circadian time (CT) was defined based on the onset of locomotor activity (averaged time in a group, CT 12)

Next, we examined the expression rhythms of clock genes under CJL in the liver and adrenal glands, which are representative of the peripheral circadian clock organs. The expression of core circadian clock genes (*Bmal1*, *Per1*, *Per2*, *Cry1*, and *Rev-erbα*) and a clock-controlled gene (*Dbp*) were analyzed. The overall expression of *Per2* (*p* = 0.0434) in females and *Dbp* (*p* = 0.0193) in males was suppressed by CJL treatment (Fig. 3). The rhythmicity of clock genes was also evaluated using cosinor analysis (Table 1). Following CJL treatment, the significant rhythmicity of *Bmal1*, *Per1*, *Per2*, and *Cry1* expression was abolished in female mice. Conversely, in male mice, only the expression of *Bmal1* lost significant rhythmicity. These results demonstrated a significant sex-dependent effect of CJL on peripheral circadian clocks. Similar results were observed in the adrenal gland, where significant rhythmicity in clock gene expression was more severely abolished in females than males, and rhythm amplitude of *Dbp* expression was 52% and 95% in CJL female and male mice, respectively, compared to that of controls (Supplementary Fig. 3 and Supplementary Table 2).

**Table 1:** Evaluation of circadian rhythmicity of clock genes expression in the liver by cosinor analysis

Female-liver						
	Control			CJL		
Gene	Acrophase	Amplitude	*p*-value	Acrophase	Amplitude	*p*-value
*Bmal1*	20.107	9.293	< 0.001	14.667		n.s.^1)^
*Per1*	11.282	1.856	0.006	1.253		n.s.
*Per2*	13.150	8.742	< 0.001	23.146		n.s.
*Cry1*	17.884	2.114	< 0.001	17.401		n.s.
*Dbp*	8.272	4.840	< 0.001	1.988	3.245	0.024
*Rev-erbα*	4.910	15.065	< 0.001	1.985	8.996	0.009
**Male-liver**						
*Bmal1*	21.172	9.696	< 0.001	0.419		n.s.
*Per1*	12.360	5.271	< 0.001	15.481	3.609	0.002
*Per2*	14.455	3.819	< 0.001	18.394	5.331	0.011
*Cry1*	20.345	2.720	< 0.001	21.017	2.482	0.003
*Dbp*	8.929	15.082	< 0.001	12.018	7.514	0.002
*Rev-erbα*	7.285	10.549	< 0.001	9.003	10.277	0.015

1) n.s., not significant

### Sex-dependent effects of CJL on metabolic parameters in livers and plasma

**Fig. 4:**
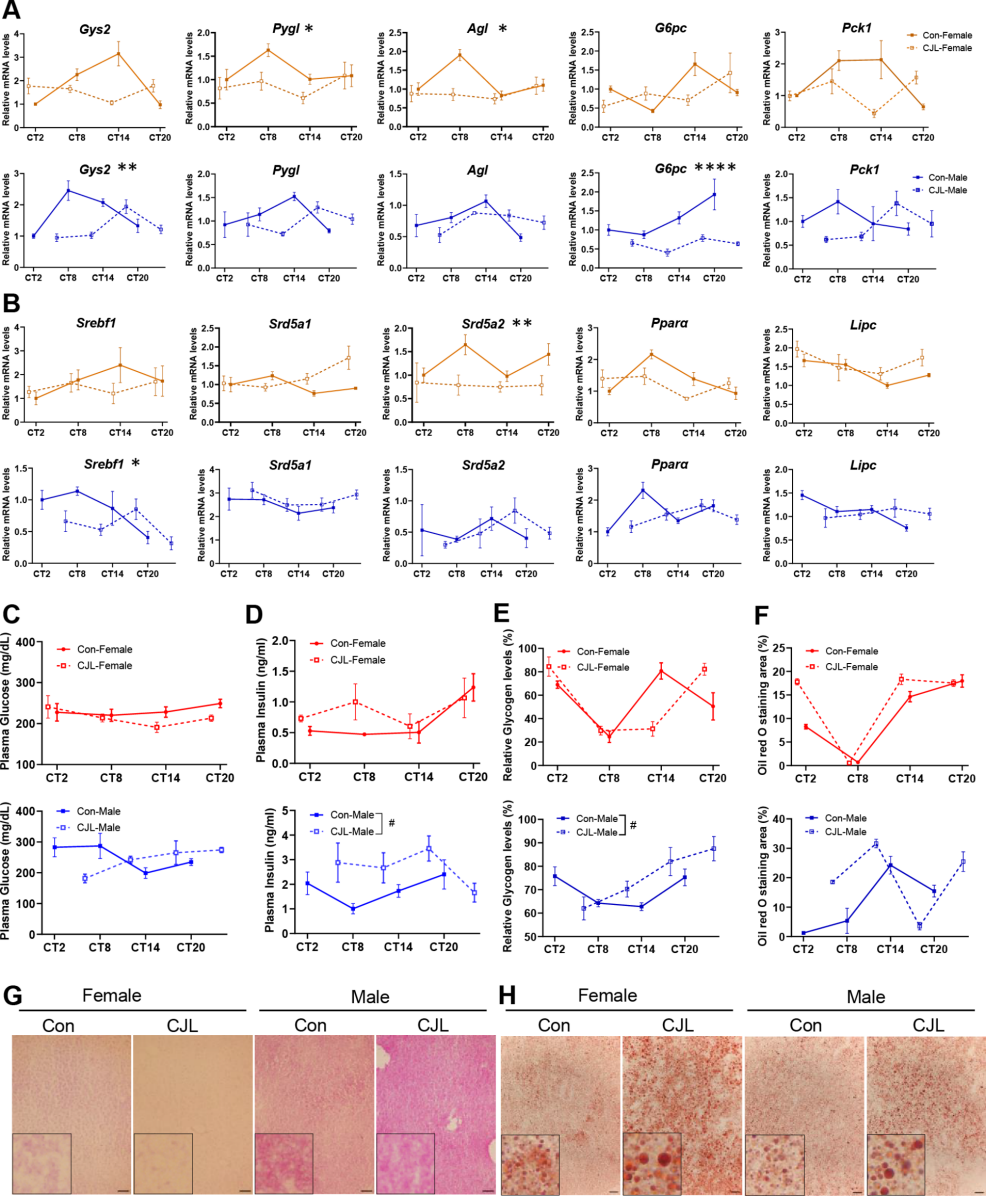
Sex-dependent effects of CJL on hepatic metabolic gene expression, plasma levels of glucose and insulin, and liver glycogen and fat accumulation. (**A**, **B**) Expression of the circadian clock-controlled glucose metabolic genes (*Gys2*, *Pygl*, *Agl*, *G6pc*, and * Pck1*) (**A**) and lipid metabolism-associated genes (*Srebf1*, *Srd5a1*, *Srd5a2*, *Pparα*, and *Lipc*) (**B**) expression in the liver of female and male C57BL/6N mice in control or chronic jet lag (CJL) groups. Two-way ANOVA, main effects of CJL treatments, *p < 0.05, **p < 0.01, ****p < 0.0001, based on sampling time points (2:00, 8:00, 14:00, and 20:00). **(C**, **D**) Glucose (**C**) and insulin (**D**) levels in plasma during 24 h under control and CJL conditions. (**E**–**H**) Quantification of hepatic glycogen (**E**) and fat contents (**F**), and representative images of periodic acid-Shiff (PAS) staining (**G**) and Oil red O staining (**H**) in liver (sampled at 8:00; CT 8 for control female and male mice, CT 13 for CJL female mice, and CT 24 for CJL male mice). Scale bars, 50 μm for PAS staining (**G**), 20 μm for Oil red O staining (**H**). Insets, five-fold magnification. Two-way ANOVA, main effects of CJL treatments, *# p* < 0.05, based on sampling time points (2:00, 8:00, 14:00, and 20:00). Circadian time (CT) was defined based on the onset of locomotor activity (averaged time in a group, CT 12). Data are presented as means ± standard errors of the means (S.E.M) (*n* = 4)

Next, we explored whether the CJL-induced reduction in the expression rhythms of clock genes in female livers was reflected in the expression patterns of clock-controlled genes involved in glucose and lipid metabolism. Sexual dimorphism was observed in the expression patterns of several genes; however, sex dependency was more complicated than clock gene expression. Regarding genes involved in glucose homeostasis, CJL treatment significantly downregulated the expression of the glycogen synthase gene *Gys2* (*p* = 0.0021) in male mice, whereas the expression of glycogen degradation genes (*Pygl* and *Agl*) was reduced in response to CJL treatment in female mice (*p* = 0.0372 and *p* = 0.0142, respectively; Fig. 4A). Glucose-6-phosphatase (G6Pase) plays a crucial role in gluconeogenesis and glycogenolysis. The expression of *G6pc* remained largely unchanged in female mice but was significantly decreased in male mice following CJL (*p* < 0.0001). Another regulator of gluconeogenesis, *Pck1*, was relatively stable in male and female CJL mice. Regarding lipid metabolism-associated genes (Fig. 4B), the expression of the steroid metabolic gene *Srebf1* was diminished exclusively in male mice treated with CJL (*p* = 0.0395). Female CJL mice exhibited reduced expression of *Srd5a2* (*p* = 0.0066) that plays a crucial role in steroid metabolism. *Pparα*, a regulator of fatty acid oxidation, as well as *Lipc*, which encodes hepatic lipase and facilitates phospholipase A1 activity along with triglyceride lipase activity, exhibited no significant changes in both female and male mice under CJL. Cosinor analysis detected circadian rhythmicity in several gene expression in a sex-dependent manner, and effect of CJL also depends on sex (Supplementary Table 3). *Gys2* expression exhibited significant rhythmicity in both sexes, and the rhythmicity was abolished under CJL only in females (Supplementary Table 3).

As the expression of genes involved in glucose homeostasis is highly dependent upon sex, we analyzed the effect of CJL on plasma glucose, insulin, as well as glycogen and fat levels in the liver. Plasma glucose levels were not significantly affected by CJL in either male or female mice (Fig. 4C), whereas plasma insulin levels were upregulated throughout 24 h by CJL only in male mice (*p* = 0.0238, Fig. 4D). Liver glycogen levels increased under CJL in male mice but not in female mice (*p* = 0.0452), while overall accumulation of liver fat was not significantly affected by CJL in both male and female mice (Fig. 4E–H).

### Glucose intolerance with reduced insulin response under CJL in males

**Fig. 5:**
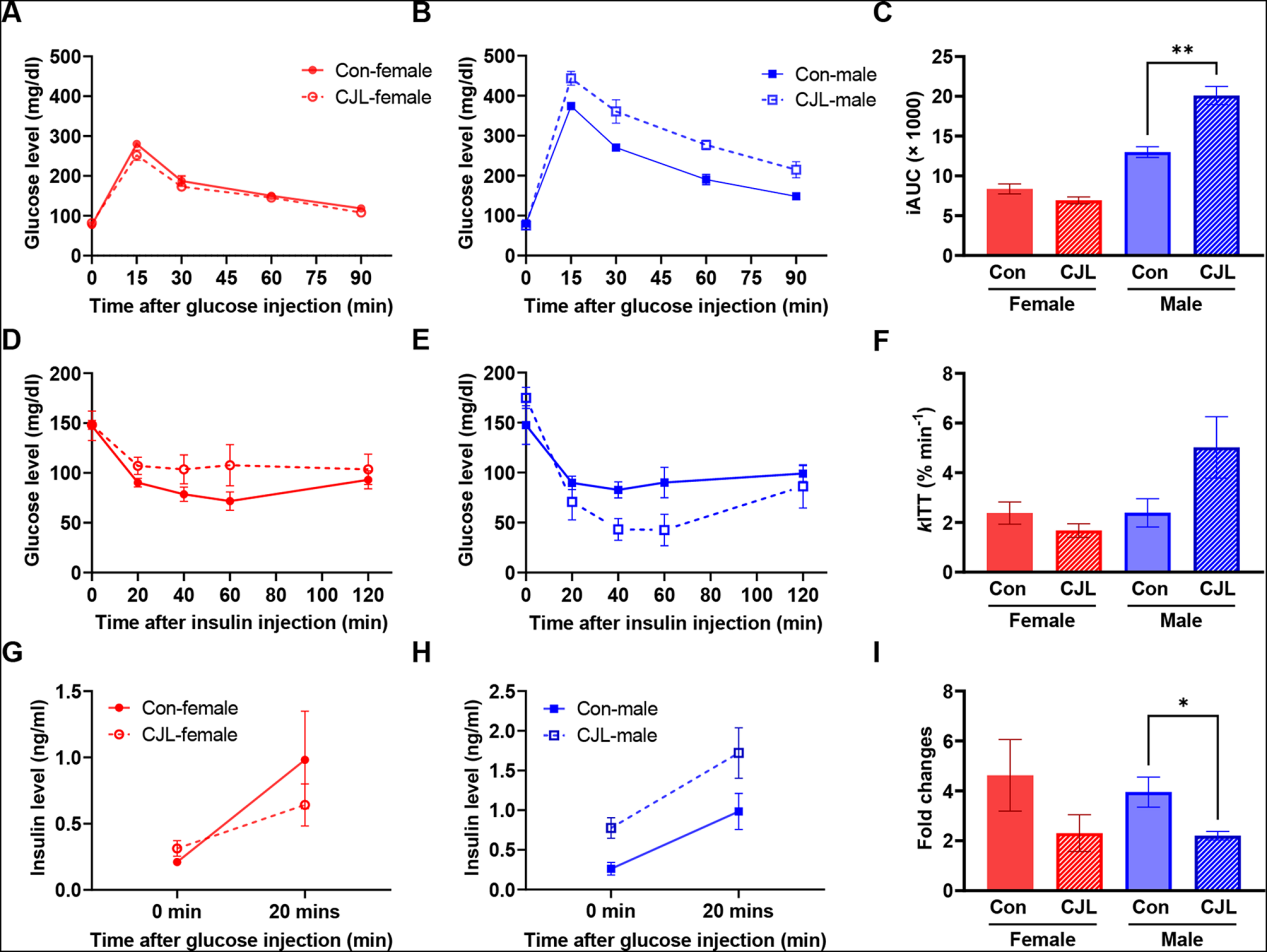
Glucose intolerance with reduced insulin response under CJL in males. (**A–C**) Results of glucose tolerance test in female (**A**) and male (**B**) C57BL/6N mice under control and chronic jet lag (CJL) conditions, and incremental area under curve (iAUC) of them (**C**). Changes of blood glucose levels in response to intraperitoneal injections of 2 g/kg glucose are shown. (**D–F**) Results of insulin tolerance test in female (**D**) and male (**E**) C57BL/6N mice under control and CJL conditions, and *k*ITT values of them (**F**). Changes in blood glucose levels in response to intraperitoneal injections of insulin (0.75 units/kg) are shown. (**G–I**) Insulin response to glucose in female (**G**) and male (**H**) C57BL/6N mice under control and CJL conditions, and fold changes of the induction. Changes in plasma insulin levels in response to intraperitoneal injections of 2 g/kg glucose are shown. *t*-test, *p < 0.05, **p < 0.01. Data are presented as means ± standard errors of the means (S.E.M) (*n* = 4)

The above data demonstrated that body weight gain under CJL, the expression rhythms of glucose metabolic genes in the liver, and plasma insulin levels were highly dependent upon sex. Therefore, we hypothesized that glucose intolerance and insulin resistance develop in a sex-dependent manner. To test this hypothesis, we performed intraperitoneal glucose and insulin tolerance tests in male and female mice exposed to CJL. In the glucose tolerance test, male mice subjected to CJL exhibited a rapid and pronounced increase in glucose levels, exceeding those observed in the control group, whereas no significant differences were observed in female CJL mice (Fig. 5A and B). The iAUC was significantly increased by CJL in males (*p* = 0.003) but not in females (Fig. 5C). Insulin tolerance test results (insulin: 0.75 unit/kg) revealed no signs of insulin resistance in both sexes, despite the observed effects on glucose tolerance in male mice (Fig. 5D–F). The insulin tolerance using other concentrations of insulin (1 and 0.5 units/kg) was also tested, and similar results were observed (Supplementary Fig. 4A and 4B).

We then hypothesized that glucose intolerance in male mice is caused by a lowered response to insulin secretion after glucose injection. Although plasma insulin levels were elevated after glucose injections in both sexes under CJL conditions (Fig. 5G and H), the fold changes in elevated levels to initial levels were significantly lower in CJL males than they were in control males (*p* = 0.0492), whereas no significant changes were detected in females (Fig. 5I). These results suggest that CJL treatment caused glucose intolerance only in male mice, and this was derived from a blunted response to inulin but not insulin resistance.

### Testosterone-dependent changes in body weight, glucose intolerance, and temperature rhythm under CJL

**Fig. 6:**
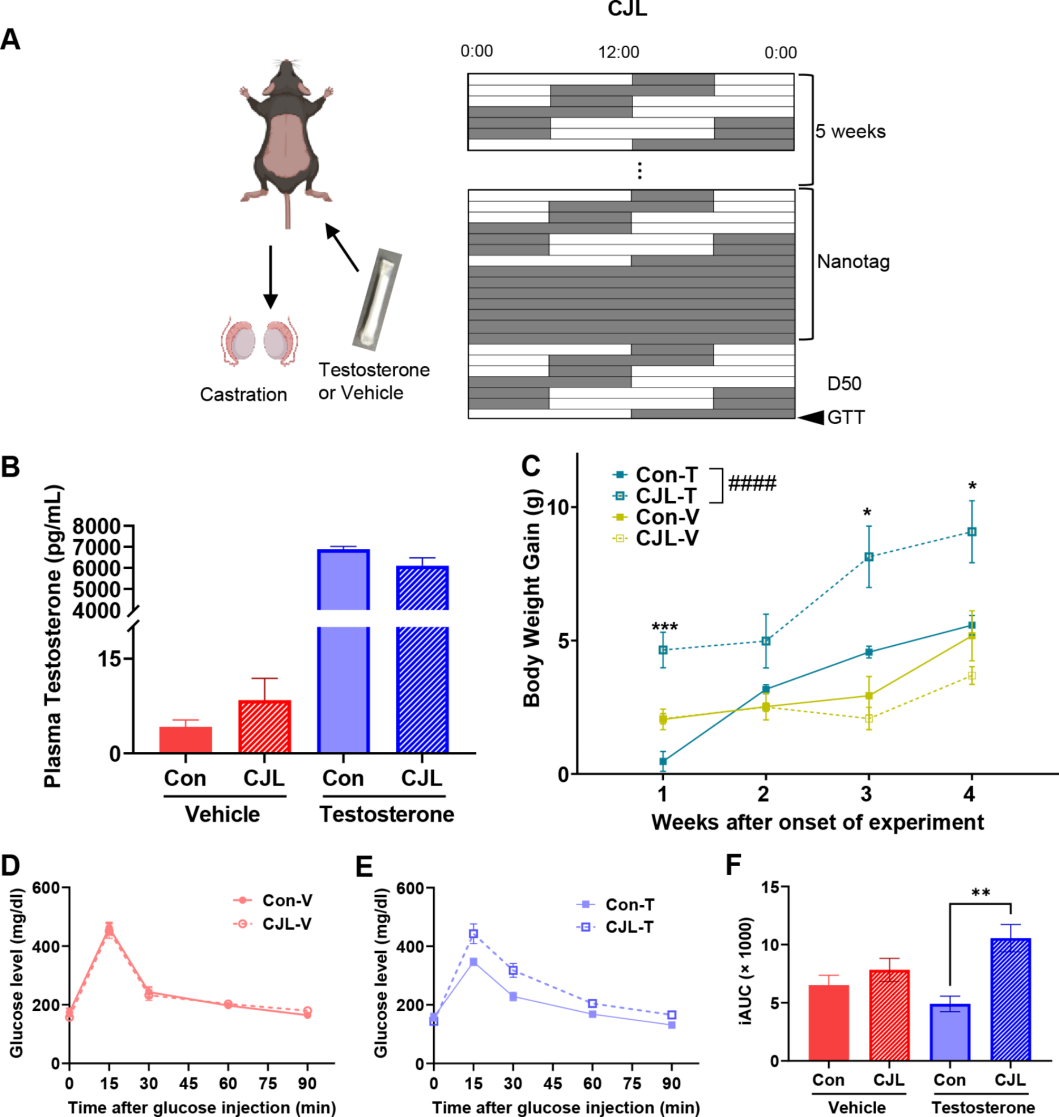
Testosterone-dependent changes in body weight and glucose intolerance in CJL males. (**A**) Castration and testosterone or vehicle replacement and the experimental schedules using male C57BL/6N mice. White and gray colors represent the light and dark phases, respectively. (**B**) Plasma testosterone levels in castrated mice with vehicle-implantation (Con-V and CJL-V) or testosterone-implantation (Con-T and CJL-T) under control (Con-V and Con-T) or chronic jet lag (CJL-V and CJL-T). (**C**) Temporal changes in body weight gain under CJL treatment. (**D–F**) Results of glucose tolerance test in castrated mice with vehicle-implantation (**D**) and testosterone-implantation (**E**), and the incremental area under curve (iAUC) of them (**F**). Changes of blood glucose levels in response to intraperitoneal injections of 2 g/kg glucose are shown. *t-*test, * p < 0.05. Data are presented as means ± standard errors of the means (S.E.M) (*n* = 5)

To elucidate the underlying mechanisms contributing to the observed sex-specific differences in the response to CJL, we focused on the role of testosterone that exerts a critical effect on insulin secretion and glucose homeostasis [33]. We performed castration and testosterone- or vehicle-containing tubing implantation in mice and subjected them to control (12 L: 12D) and CJL conditions (Fig. 6A). Implantation of testosterone tubing in castrated mice resulted in physiological levels of plasma testosterone (6–8 ng/mL), while castrated mice with vehicle implantation exhibited substantially low levels (Fig. 6B). In the cohort of castrated mice with vehicle implantation, body weight gain was not altered by CJL (Fig. 6C). Treatment abolished male patterns and mimicked female patterns observed in intact mice (Fig. 1B). In contrast, castrated mice implanted with testosterone tubing recovered the intact male pattern of increased weight gain after CJL (*p* < 0.0001, Fig. 6C). The results of the glucose tolerance test were similar to those of the body weight test. Castrated mice implanted with the vehicle exhibited no significant difference in glucose tolerance under CJL (Fig. 6D), mirroring the response observed in intact female mice (Fig. 5A). Testosterone-implantation rescued the reduced glucose tolerance under CJL conditions (Fig. 6E and F, *p* = 0.0119), a male pattern observed in intact mice (Fig. 5B).

**Fig. 7:**
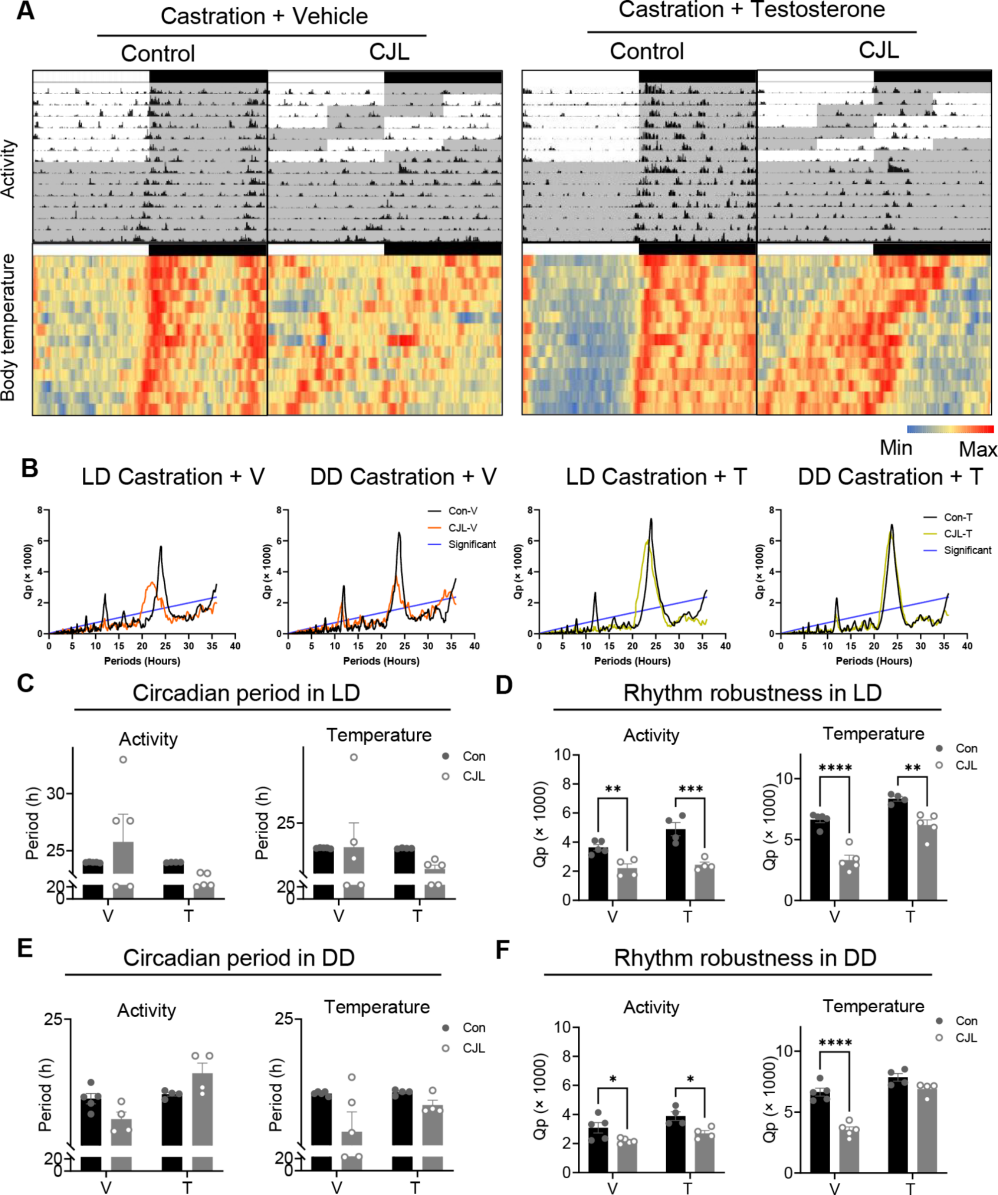
Testosterone-dependent changes in activity and temperature in CJL males. (**A**) Actograms of locomotor activity and body temperature rhythms under control or chronic jet lag (CJL) conditions in castrated mice with vehicle-implantation (V) or testosterone-implantation (T). (**B**) Chi-square periodogram based on body temperature rhythms under light-dark cycles (LD, control or CJL conditions) or following constant darkness (DD). The blue line is the significance level (*p* = 0.001). (**C–F**) The circadian period (**C, E**) and rhythm robustness (**D, F**) of activity and temperature rhythms in castrated mice with vehicle-implantation (V) or testosterone-implantation (T) under control or CJL conditions (**C, D**) or following DD (**E, F**), based on chi-square periodogram. Data are presented as means ± standard errors of the means (S.E.M). Two-way ANOVA followed by Šidák’s multiple comparison test, ** p < 0.01, *** p < 0.001, **** p < 0.0001

Activity and core body temperature rhythms were examined in these animals. Under control or CJL conditions, castrated mice with vehicle implantation did not exhibit significant prolongation of intact female mice (Fig. 7A–C). The rhythm robustness of activity and temperature decreased in castrated mice with vehicle implantation (*p* = 0.0077 and *p* < 0.0001, respectively) and in those with testosterone-implantation (*p* = 0.0001 and *p* = 0.0015, respectively; Fig. 7D). Under DD conditions, in castrated mice with vehicle implantation as well as those with testosterone-implantation, the period of activity and core body temperature maintained similar rhythmicity under CJL (Fig. 7E). However, vehicle-treated castrated mice exhibited a significant reduction in the rhythm robustness of body temperature fluctuations (Fig. 7F, *p* < 0.0001) that mirrored that of intact female mice following CJL (Fig. 2F). Testosterone replacement in castrated mice resulted in a recovered robustness of temperature rhythms under CJL, a response that was congruent with that of intact male mice (Fig. 2F).

## Discussion

Circadian misalignment is a known risk factor for metabolic disorders; however, associations with sex differences have been controversial in both humans and animals. In this study, we demonstrated significant sex-specific differences in the metabolic conditions and circadian rhythms following CJL treatment in C57BL/6N mice fed a standard diet. While circadian rhythms in locomotor activity exhibited reduced robustness after CJL in both sexes, robustness of body temperature rhythms and circadian oscillations in peripheral clocks were more vulnerable to CJL in females than they were in males. Remarkably, the sex-dependent changes in circadian parameters were not directly reflected in metabolic changes (i.e., body weight gain increased in male mice and decreased in female mice). Our data also demonstrated that glucose intolerance, continuously elevated plasma insulin levels, reduced insulin response, and high liver glycogen levels were exclusively observed in male CJL mice. Furthermore, CJL-induced changes in clock gene expression in the liver are unlikely to account for expression patterns of clock-controlled genes involved in glucose and lipid metabolism. These data suggest that the effect of CJL on metabolic consequences is mediated by not only the circadian clock system but also by complex interactions between the circadian clock, metabolic hormones, stress response, and sex steroid-related pathways. According to our hypothesis, the reduction in body weight in female mice may be related to their high sensitivity to stress [34], given that anxiety-like behavior is induced by CJL only in female mice. In contrast, body weight gain in CJL males is likely linked to glucose regulation mechanisms that are sensitive to testosterone and its metabolites [35–37]. Castration and testosterone replacement experiments suggest that testosterone regulates CJL-induced body weight and glucose intolerance changes as well as a resilient circadian clock that controls body temperature rhythms. These findings highlight the significant role of testosterone in regulating metabolic conditions and sustaining a robust temperature rhythm under circadian misalignment.

Our study primarily used C57BL/6N mice in the CJL experiments after comparison with C57BL/6J mice. We demonstrated that sex differences were more pronounced in C57BL/6N mice, suggesting that this strain is a preferable choice for studying circadian misalignment under a standard diet. In the field of the circadian clock, C57BL/6J is a popular strain, and accumulated data have been published. However, C57BL/6N and C57BL/6J mice exhibit discrepancies in their genetic backgrounds [38]. For example, the C57BL/6J strain has a spontaneous mutation in the nicotinamide nucleotide transhydrogenase gene, which may lead to reduced glucose tolerance [39]. Responsiveness to HFD-induced obesity also differs between substrains. C57BL/6J is more vulnerable than the C57BL/6N strain, as evidenced by several studies [28, 29]. Additionally, behavioral responses to constant light, running-wheel access, shortened light-dark cycles, and many other phenotypes, including pain sensitivity that is highly dependent on gonadal hormones [40], differ between these substrains [38, 41]. Notably, male C57BL/6N mice exhibit pronounced body weight gain under constant light [41], suggesting that metabolism in C57BL/6N mice is likely to be more sensitive to disturbed lighting conditions than in C57BL/6J mice.

In the present study, we demonstrated sustained rhythms of activity, temperature fluctuations, and clock gene expression in C57BL/6N male mice subjected to CJL treatment (6 h advance of the light-dark cycle every two days). Conversely, female mice exhibited rhythms that were more susceptible to these parameters under CJL or DD after CJL. We observed a long-period (25.0–26.9 h) of activity and body temperature rhythms in female mice under CJL. A previous study by Casiraghi et al. [14] demonstrated desynchronization of activity rhythms in male C57BL/6 mice subjected to CJL (6 h advances of light-dark cycles every 2 days). Several mice exhibited both short- and long-period components (approximately 21.0 h and 24.7 h), while several mice exhibited one of these components. Although we did not observe mixed components, our data may indicate desynchronized conditions only in females. However, a previous study [42] demonstrated that male and female albino mice under CJL (9 h advance of the light-dark cycle every two days) did not exhibit significant differences in activity. Furthermore, circadian rhythms were significantly dampened by CJL in both sexes. A recent study using C57BL/6J mice on a HFD reported that behavioral and transcriptional rhythmicity in female mice is more resilient than that in males under chronic circadian misalignment light conditions (8 h advance of the light-dark cycle once a week) [26], which is substantially different from our data. These data suggest that the vulnerability of the circadian clock and metabolic conditions to circadian misalignment depends on dietary conditions, genetic background, and light-shifting schedules. In line with this assumption, a HFD worsens CJL-induced glucose intolerance and increases the body weight in male mice [43]. To understand the effects of HFD and genetic background on sex dependency, CJL experiments using both C57BL/6 substrains should be performed under a HFD in the future.

A human study focusing on shift workers reported less nutritionally dense diets with a lower intake of fruits and vegetables and higher consumption of foods high in free sugars, saturated fats, and caffeinated beverages [44]. Another study also demonstrated that under a circadian misalignment protocol (a rapid 12 h shift of the behavioral/environmental cycle for three test days), appetite perceptions differed between males and females, where male subjects reported an increase in cravings for energy-dense and savory foods compared to females [21]. These studies suggest that under conditions of CJL, distinct food preferences emerge between males and females. Such food preferences may be related to sex-dependent metabolic changes under the circadian misalignment observed in our study. Additionally, stress and sleep disturbances that may accompany CJL treatment may impact food choices in a sex-dependent manner [45]. In a previous study, social jet lag combined with a cafeteria diet induced overconsumption and the development of obesity and metabolic syndrome in rats [46]. Our study suggests that a standard diet may prevent male mice from being exposed to a vulnerable circadian clock under CJL, but not to glucose intolerance or overweight.

Through the analysis of circadian clock gene expression in the liver and adrenal gland, we demonstrated the susceptibility of peripheral clocks to circadian misalignment accompanied by vulnerable activity and body temperature rhythm changes in female mice. However, the altered patterns of clock genes were only partially reflected in the expression of metabolic genes involved in glucose and lipid metabolism. *Gys2* is a clock-controlled gene [5], and the expression rhythms in the liver were abolished by CJL only in females, which is in line with the susceptibility of the peripheral clocks in them. Conversely, *G6pc* is regulated by the molecular clockwork [47] as well as by diverse metabolic and hormonal signals, including insulin, that suppress glycogenolysis. High insulin levels in the plasma of CJL males suggest that insulin is a dominant factor in regulating *G6pc* expression. Other metabolic genes are also regulated by both the circadian clock and hormonal signals. Thus, these data suggest that many metabolic genes are under circadian regulation, but this regulation is modulated or summed up by hormonal signals in a gene-specific manner.

The overall expression levels of *Gys2* were remarkably decreased by CJL in male mice, along with a reduction in *Dbp* expression. These results are consistent with observations in *Clock* mutation mice [5] that exhibit phenotypes of obesity and impaired glucose tolerance [48, 49]. *G6pc* expression was significantly suppressed by CJL treatment in male mice, indicating impaired gluconeogenesis and glycogenolysis. This was corroborated by elevated liver glycogen levels in CJL-treated male mice in the present study, mirroring the results observed in *G6pc* mutant mice [50]. In females, the expression of glycogen degradation-related genes *Pygl* and *Agl* was significantly reduced by CJL. However, unlike in males, liver glycogen levels did not exhibit significant changes in CJL in females. The alterations observed in these genes may be orchestrated by the modulation of circadian clock expression operating through intricate pathways. These findings suggest that under conditions of CJL, male and female mice regulate glucose and lipid metabolism via distinct pathways involving different regulatory genes.

In the present study, only males exhibited a significant reduction in glucose tolerance along with a weakened insulin response observed according to the insulin response test when subjected to CJL. These findings were consistent with those of numerous studies involving human subjects [10, 26, 51]. However, insulin resistance was not observed in the insulin tolerance test. The insulin response to glucose was slightly blunted by CJL in males, suggesting that insulin secretion from the pancreas was impaired by CJL. A previous study reported that 10-week circadian rhythm disturbances by 6 h advances of light-dark cycles every 3 days or constant light accelerated the loss of beta cell function in diabetes-prone human islet amyloid polypeptide transgenic rats [52]. In another study, several pancreas-specific genes related to insulin secretion displayed a concomitant phase shift in expression after CJL exposure [53]. These alterations may blunt pancreatic insulin secretion. However, plasma insulin levels were marginally elevated in male mice under CJL conditions and remained unchanged in female mice. Constitutive upregulation of plasma insulin levels without insulin resistance or hypoglycemia suggests that insulin clearance is also impaired in males with CJL. Insulin clearance exhibits diurnal variations in humans [54]. These variations are likely mediated by the circadian clock, given that *Per2*-deficient mice exhibit elevated plasma insulin levels and impaired insulin clearance [55]. Glucose intolerance and normal insulin sensitivity in male mice under CJL suggest that not only the insulin response to glucose but also the glucose disposal process is inhibited under CJL conditions via insulin-independent glucose disposal mechanisms [56, 57]. These mechanisms contribute to 70% of glucose disposal after glucose injection, referred to as glucose effectiveness [58], and are involved in the brain, nervous system, red blood cells, and other insulin-independent tissues/organs. Previous studies have demonstrated that two incretin hormones, glucose-dependent insulinotropic polypeptide and glucagon-like peptide-1, increase insulin-independent glucose disposal [56]. The validation of these hypotheses could provide a novel perspective on the metabolic effects of CJL.

Our study focused on the role of testosterone, but not of estradiol, in the sex-dependent effects of CJL. The roles of the ovary and estradiol have previously been demonstrated to exert activation effects on the temporal patterning and expression of daily and circadian behavior [18] and on maintaining normal glucose tolerance and insulin response [59]. Ovariectomized mice exhibited impaired re-entrainment to circadian misalignment and low-amplitude rhythms in *Bmal1* but no rhythms in *Per2*, as demonstrated in a previous study [26] under a HFD. Thus, a study focusing on the role of the ovary and estradiol in C57BL/6N mice fed a standard diet is necessary to fully elucidate the mechanisms underlying sex differences in CJL.

In summary, our findings shed light on the sex-dependent effects of CJL on both circadian rhythm and metabolism in C57BL/6N mice fed a standard diet. Testosterone plays a key role in maintaining the circadian clock and regulating metabolism during CJL. The intricate pathways involved in sex-differentiated metabolic regulation are yet to be fully elucidated, indicating a vast and promising field for future research. This research paves the way for novel sex-dependent therapeutic strategies and preventive measures to manage circadian clock disruption and associated metabolic disorders. Further research is required to validate these findings and fully elucidate the complex interplay between sex hormones, circadian rhythms, and metabolic processes.

## Electronic supplementary material

Below is the link to the electronic supplementary material.

Supplementary Material 1

## Data Availability

The datasets used and/or analyzed in the current study are available from the corresponding author upon reasonable request.
