# Supplementary material for: Sex-dependent effects of chronic jet lag on circadian rhythm and metabolism in mice
Source: Biol Sex Differ. 2024 Dec 5;15:102. doi: 10.1186/s13293-024-00679-z (PMC11619446; doi:10.1186/s13293-024-00679-z)
Supplement: Supplementary file 1 — Supplementary Material 1 [file 13293_2024_679_MOESM1_ESM.pdf]

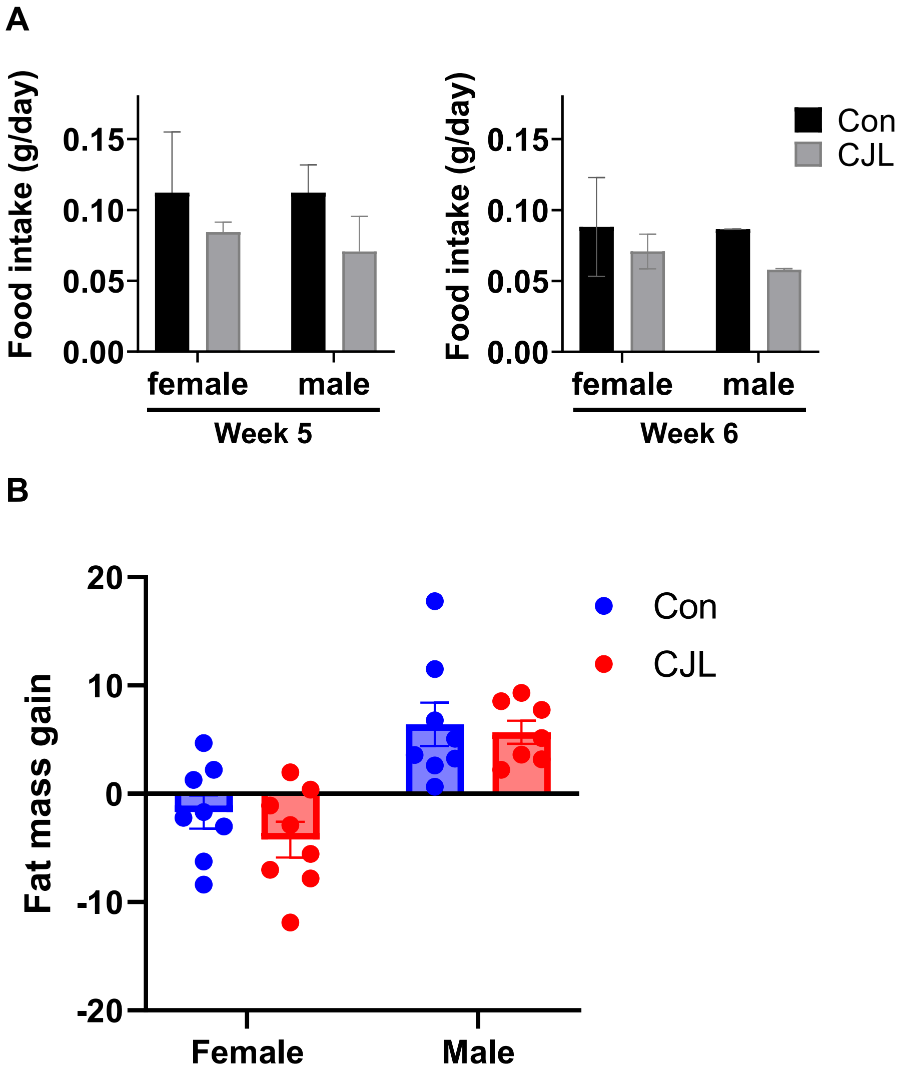

Supplementary Figure 1. Effect of CJL on food intake and fat mass in C57BL/6N mice.
A) Food intake per mouse for one day recorded at week 5 and week 6 under control or CJL treatment, normalized by body weight (per gram). B) Fat mass gain during 6 weeks of control or CJL treatment. Data are presented as means ± standard errors of the means (S.E.M) (n = 8)

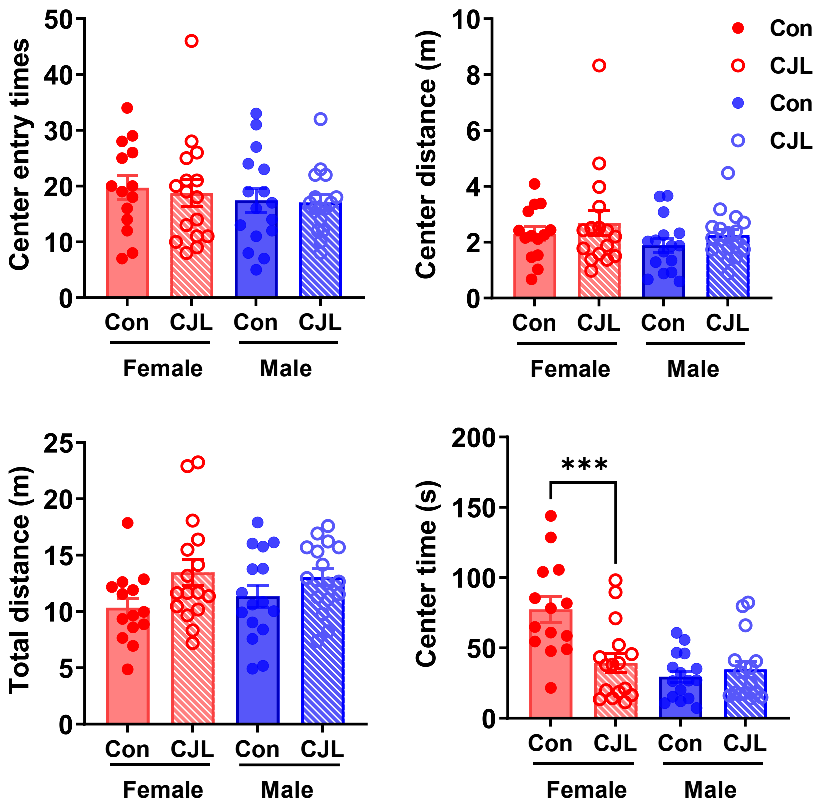

Supplementary Figure 2. Effect of CJL on open filed behaviors in C57BL/6N mice
The center entry times, center distance, total distance, and center time detected in the open filed test under control or CJL treatment. t-test, *** $p < 0.001$. Data are presented as means ± standard errors of the means (S.E.M) (n = 14-16)

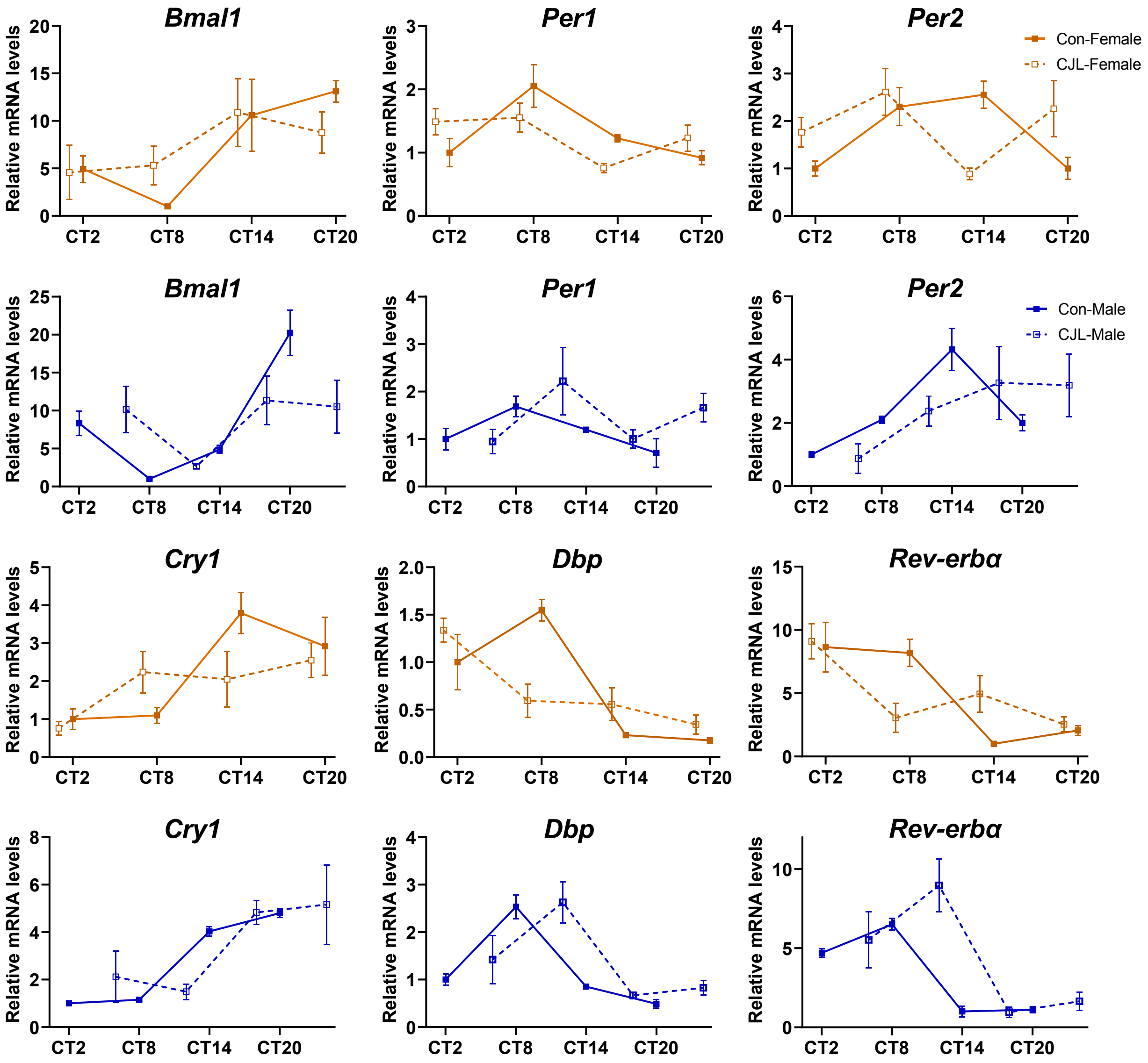

Supplementary Figure 3. Effect of CJL on temporal expression of clock genes in the adrenal gland.

Expression of the circadian clock genes *Bmal1*, *Per1*, *Per2*, *Cry1* and *Rev-erbα* and clock-controlled gene (*Dbp*) in female and male C57BL/6N mice under control or CJL treatment. Data are presented as means ± standard errors of the means (S.E.M) (n = 4).

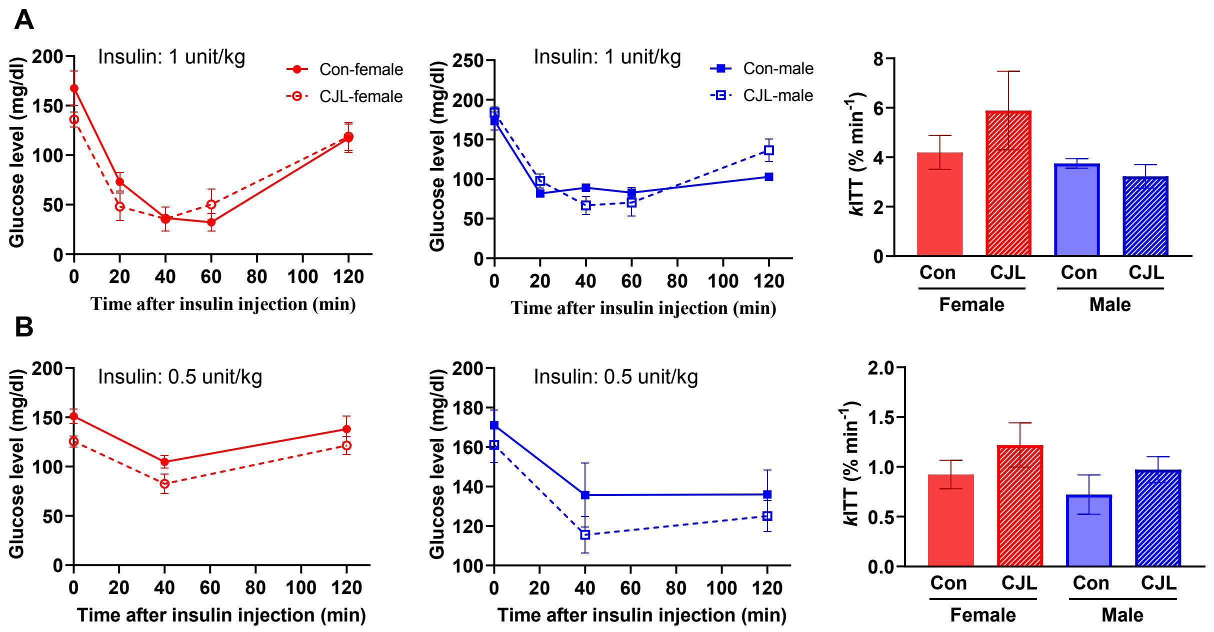

Supplementary Figure 4. Effect of CJL on insulin tolerance in C57BL/6N mice.
Changes in blood glucose levels in response to intraperitoneal injections of insulin (A: 1 unit/kg, B: 0.5 unit/kg) in female and male mice under control or CJL treatment, and the *k*ITT value of them. Data are presented as means ± standard errors of the means (S.E.M) (n = 4).

Supplementary Table 1. Primer sequences used for qPCR

Gene	GeneBank No.	Sequence 5' - 3'	Amplicon (bp)
mDbp	NM_016974.4	F: CCGTGGAGGTGCTAATGACCT<br>R: CCTCTGAGAAGCGGTGTCT	105
mBmal1	NM_001357070.2	F: GCAGTGCCACTGACTACCAAGA<br>R: TCCTGGACATTGCATTGCAT	201
mPer1	NM_011065.5	F: AGAAGAAAACAGCACCAGCT<br>R: TCTTGAGTTATAAGAACCCCAACATG	98
mPer2	NM_001420881.1	F: GCCAAGTTTGTGGAGTTCCTG<br>R: CTTGCACCTTGACCAGGTAGG	226
mRev-erbα	NM_145434.4	F: CCCTGGACTCCAATAACAACACA<br>R: GCCATTGGAGCTGTCACTGTAG	110
mCry1	NM_001428427.1	F: GGATCCACCATTTAGCCAGACAC<br>R: CATTTATGCTCCAATCTGCATCAAG	126
mGys2	NM_145572.3	F: CCAGACAAATTCCACCTAGAGC<br>R: GGGCCTGGGATACTTAAAGC	66
mG6pc	NM_008061.4	F: TGGTAGCCCTGTCTTTCTTTG<br>R: TTCCAGCATTCACACTTTCCT	90
mPck1	NM_011044.3	F: GTGGGCGATGACATTGCC<br>R: ACTGAGGTGCCAGGAGCAAC	101
mSrebf1	NM_001358314.1	F: ATCGGCGCGGAAGCTGTCGGGGTAGCGTC<br>R: ACTGTCTTGGTTGTTGATGAGCTGGAGCAT	116
mPparα	NM_011144.6	F: TGCAAACTTGGACTTGAACG<br>R: AGGAGGACAGCATCGTGAAG	80
mLipc	NM_001411789.1	F: TGAGCACCAAGAAGCACTCT<br>R: CTCCCAAAGGGCTCTGTTCC	177
mHprt	NM_013556.2	F: TGAGCACCAAGAAGCACTCT<br>R: CTCCCAAAGGGCTCTGTTCC	111
m36b4	NM_007475.5	F: CTCACTGAGATTCGGGATATG<br>R: CTCCCACCTTGTCTCCAGTC	223
mPygl	NM_133198.2	F: CCCCGTGCCTGGATATATGA<br>R: TGTTTCAGCCGCAACTCCTT	201
mAgl	NM_001362367.1	F: GGCATCACCGGCAGATACA<br>R: GGAGCCTATTGATGGCACATC	77
mSrd5a1	NM_175283	F: AGTGGTGTGGCTTTGCACTG<br>R: TCTCGAGGTACCACTGATGATG	114
mSrd5a2	NM_053188	F: GATCCTGTGCTTTGGGAAACC<br>R: GCATCCCTACCGACACCAC	134

Supplementary Table 2. Evaluation of circadian rhythmicity of clock genes expression in the adrenal gland by cosinor analysis.

Female-adrenal gland

	**Con**			**CJL**		
**Clock**	**Acrophase**	**Amplitude**	***p*-value**	**Acrophase**	**Amplitude**	***p*-value**
Bmal1	18.332	6.690	0.002	14.915		n.s. [1)]
Per1	8.751	0.578	0.011	2.585	0.399	0.042
Per2	11.342	1.014	0.006	2.462		n.s.
Cry1	16.203	1.667	0.001	13.798		n.s.
Dbp	6.047	4.485	< 0.001	2.183	2.338	0.032
Rev-erbα	4.583	4.898	< 0.001	1.472		n.s.
**Male-adrenal gland**						
Bmal1	20.904	9.469	< 0.001	23.421		n.s.
Per1	9.644	0.498	0.016	11.232		n.s.
Per2	13.888	1.662	< 0.001	19.254		n.s.
Cry1	17.353	2.373	< 0.001	13.570	2.283	0.022
Dbp	7.724	2.096	< 0.001	10.488	1.992	0.011
Rev-erbα	5.700	3.274	< 0.001	9.866	4.315	0.002

Supplementary Table 3. Evaluation of circadian rhythmicity of metabolic genes expression in the liver by cosinor analysis.

Female-liver

	**Control**			**CJL**		
**Gene**	**Acrophase**	**Amplitude**	***p*-value**	**Acrophase**	**Amplitude**	***p*-value**
Gys2	11.949	1.253	0.003	0.308		n.s.
Pygl	8.067		n.s	22.991		n.s.
Agl	7.200		n.s	21.060		n.s.
G6pc	16.430		n.s.	17.886		n.s.
Pck1	10.899	0.486	0.005	0.180		n.s.
Srebf1	13.871		n.s.	22.485		n.s.
Srd5a1	5.645		n.s.	18.425		n.s.
Srd5a2	7.590		n.s.	1.128		n.s.
pparα	9.161	0.042	< 0.001	6.092	0.016	0.041
Lipc	3.506	0.359	0.003	23.526		n.s.

Male-liver

Gys2	10.904	0.778	0.008	18.705	0.506	0.004
Pygl	11.997	0.378	0.039	20.722		n.s.
Agl	11.365	0.371	0.045	16.227		n.s.
G6pc	18.887	0.146	0.018	22.073	2.482	0.003
Pck1	7.689		n.s.	19.284	0.172	0.037
Srebf1	8.526	0.072	0.031	14.766		n.s.
Srd5a1	3.975		n.s.	3.596		n.s.
Srd5a2	14.362		n.s.	18.025		n.s.
pparα	10.375		n.s.	17.092	0.016	0.041
Lipc	5.240		n.s.	18.202		n.s.

1) n.s., not significant
